# Behavioural susceptibility theory: the role of appetite in genetic susceptibility to obesity in early life

**DOI:** 10.1098/rstb.2022.0223

**Published:** 2023-09-11

**Authors:** C. H. Llewellyn, A. R. Kininmonth, M. Herle, Z. Nas, A. D. Smith, S. Carnell, A. Fildes

**Affiliations:** ^1^ Research Department of Behavioural Science and Health, Institute of Epidemiology and Health Care, University College London, London, WC1E 6BT, UK; ^2^ Social, Genetic & Developmental Psychiatry Centre, Institute of Psychiatry, Psychology & Neuroscience, King's College London, London, SE5 8AF, UK; ^3^ MRC Epidemiology Unit, University of Cambridge, Cambridge, CB2 0SL, UK; ^4^ Department of Psychiatry and Behavioural Sciences, Johns Hopkins University School of Medicine, Baltimore, MD21287, USA; ^5^ School of Psychology, University of Leeds, Leeds, LS2 9JT, UK

**Keywords:** behavioural susceptibility theory, genes, environment, appetite, obesity

## Abstract

Excess weight gained during the early years and, in particular, rapid weight gain in the first 2 years of life, are a major risk factors for adult obesity. The growing consensus is that childhood obesity develops from a complex interaction between genetic susceptibility and exposure to an ‘obesogenic’ environment. Behavioural susceptibility theory (BST) was developed to explain the nature of this gene–environment interaction, and why the ‘obesogenic’ environment does not affect all children equally. It hypothesizes that inherited variation in appetite, which is present from birth, determines why some infants and children overeat, and others do not, in response to environmental opportunity. That is, those who inherit genetic variants promoting an avid appetite are vulnerable to overeating and developing obesity, while those who are genetically predisposed to have a smaller appetite and lower interest in food are protected from obesity—or even at risk of being underweight. We review the breadth of research to-date that has contributed to the evidence base for BST, focusing on early life, and discuss implications and future directions for research and theory.

This article is part of a discussion meeting issue ‘Causes of obesity: theories, conjectures and evidence (Part I)’.

## 1. Background: childhood obesity develops from complex gene–environment interactions beginning in early infancy

The World Health Organisation (WHO) declared the high and rising prevalence of obesity a ‘global pandemic’ nearly 20 years ago. Since then, rates have continued to increase [1]. Of particular concern is the high prevalence in childhood. In England in 2021, approximately 1 in 10 children had developed obesity by the time they started primary school at 4–5 years of age, rising to 1 in 5 by the end of primary school at age 10–11. Once developed in childhood, obesity is difficult to reverse, with strong tracking into adolescence and adulthood [2]. What is more, weight development during the infancy period is especially important; rapid weight gain in the first 2 years of life (an upward crossing of ≥0.67 weight *z* score) is associated with a nearly 4 times increased risk of overweight or obesity in childhood or adulthood [3]. Understanding the causes of excess weight gain as early as possible in the lifespan is a public health priority.

The obesity epidemic is often attributed to major changes in the food and physical activity environments that promote positive energy imbalance. In high-income countries there have been substantial increases in the availability of highly palatable, energy-dense foods that are cheap and heavily incentivised through market forces, with children often the target [4]. At the same time, societal changes such as increased reliance on transportation have decreased energy expenditure across the population [5]. These changes have led to what is often termed an ‘obesogenic’ environment—one that encourages higher energy intake relative to expenditure. Recent generations who were born after the onset of the obesity epidemic have been exposed to its effects their entire lives. However, despite the ubiquity of the ‘obesogenic’ environment, there is large variation in body weights—and rates of weight gain—of infants and children, observable even among siblings sharing their home. The causes of obesity are more complex than the ‘obesogenic’ environment alone.

The consensus among researchers is that genetic susceptibility to the environment contributes importantly to the variability in weight. Much of what we know about genetic influence on human body weight comes from nearly a century of twin studies—a powerful design for disentangling the relative contribution of genetic and environmental factors to variation in a measured phenotype (such as weight). A meta-analysis of 45 twin cohorts from 20 countries (*n* = 87 782 pairs), spanning infancy to late adolescence, estimated that genetic variation (termed ‘heritability’) explains approximately 40–85% of individual differences in weight during the formative years [6]. Another key observation from twin studies is the low heritability of birthweight relative to the high heritability of rate of weight gain during early postnatal life, indicating that genetic influence on rate of weight gain and obesity risk starts to be expressed soon after birth [7].

Since the turn of this century, molecular genetic studies have started to elucidate obesity risk at a genomic level. Genome-wide association studies have identified many of the common genetic variants involved, which can be aggregated into polygenic risk scores (PRS). PRS quantify genetic susceptibility to higher or lower body mass index (BMI) on a continuum, using measured genomic risk across all available variants. A recent landmark study found that a PRS for adult BMI, incorporating 2.1 million genetic variants, was not strongly associated with birthweight but started to be expressed from early infancy, strengthening during childhood and adolescence, with a 12 kg difference in body weight between those in the top and bottom 10% of genomic risk by age 18 years [8]. Other important advances have come from whole genome sequencing that incorporates rare as well as common forms of genetic variation; a recent study used whole genome sequencing modelling to estimate the heritability of BMI as 30% [9]. A key question researchers have been interested in answering is *how* genes influence weight and confer differential susceptibility to the environment—i.e. what are the gene mechanisms?

## 2. Behavioural susceptibility theory: appetite is a neurobehavioural mediator of genetic susceptibility to excessive weight gain in early life

**Figure 1.**
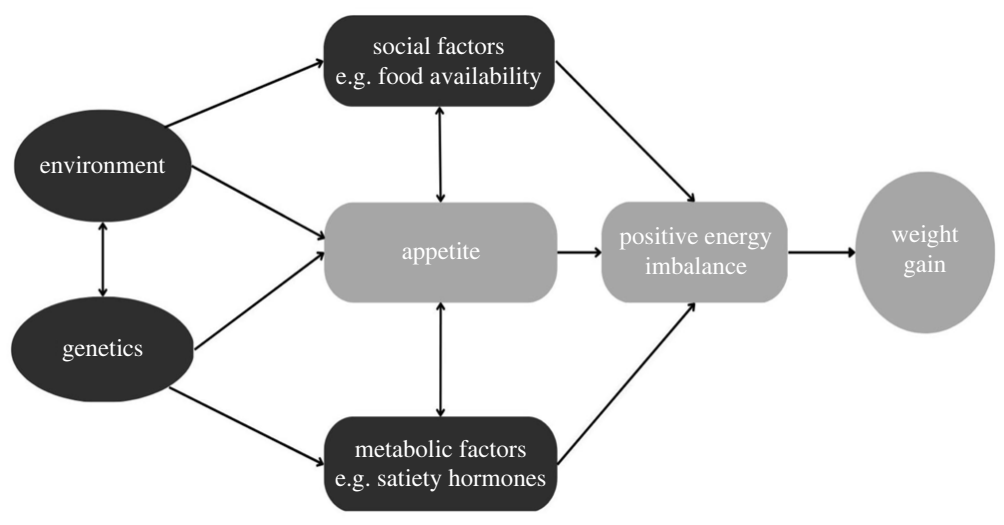
Model depicting behavioural susceptibility theory, which proposes that appetite mediates the interaction between genetic susceptibility to obesity and exposure to an obesogenic environment.

The late Professor Jane Wardle developed Behavioural Susceptibility Theory, which hypothesizes that genetic susceptibility to obesity is mediated by neurobiological processes controlling appetite regulation via gut hormones and the central nervous system, expressed as feeding or eating behaviours. Two neurologically dissociable aspects of appetite are thought to be involved: responsiveness to food cues (e.g. wanting to eat in response to the sight, smell or taste of food), which reflects hedonic processes involved in pleasure and reward; and sensitivity to internal satiety signals (e.g. feelings of fullness), which reflects homeostatic processes involved in energy balance. The central thesis is that inherited variation in these aspects of appetite confers differential susceptibility to the ‘obesogenic’ environment. Infants and children who are genetically predisposed to high ‘food responsiveness’ or weak satiety sensitivity are more vulnerable to overeating and developing obesity in an environment where food cues are pervasive, opportunities to eat are plentiful and portion sizes are large (figure 1). In contrast, those who are genetically predisposed to high satiety sensitivity or low interest in food are protected from obesity, or even at risk of underweight, under the same environmental pressures to eat.

In 2001, Wardle developed the first comprehensive psychometric measure of children's appetite, the Child Eating Behaviour Questionnaire (CEBQ) [10], to measure appetite reliably in large samples of infants and children, at low-cost. The parent-report measure assesses eight appetitive traits, including ‘food responsiveness’ and ‘satiety responsiveness'. It has good internal and test–retest reliability [10] and shows strong tracking from early to late childhood [11], indicating that appetitive traits are fairly stable and endure throughout childhood. It has been adapted for use in early infancy, during the period of exclusive milk feeding (the Baby Eating Behaviour Questionnaire, BEBQ) [12] and has been validated in breastfed and bottle-fed infants.

## 3. Developing the evidence base for behavioural susceptibility theory

The CEBQ and BEBQ have allowed researchers to test four hypotheses central to BST:

(1) Appetite avidity plays a role in susceptibility to excessive weight gain and obesity in early life.
(2) Appetite avidity is characterized by distinct patterns of overconsumption in early life.
(3) Appetite has a strong genetic basis and mediates genetic influence on weight in early life.
(4) There is gene–environment interaction in weight development in early life.

BST has been supported by a burgeoning research base spanning different study designs from experimental research, epidemiology and genetics, offering strong triangulation of evidence.

### (a) Hypothesis 1—appetite avidity plays a role in susceptibility to excessive weight gain and obesity in early life

A growing number of studies have examined cross-sectional and prospective associations between ‘food responsiveness’ and ‘satiety responsiveness' measured using the BEBQ and CEBQ, and adiposity outcomes in infancy and childhood, with some employing innovative designs. Studies have been undertaken in large population-based cohorts, included participants from birth to late adolescence, and span Europe, North and South America, Asia and Australasia.

A comprehensive systematic review and meta-analysis of 72 studies quantified cross-sectional associations between adiposity and these appetitive traits in childhood and examined directionality of prospective associations [13]. Cross-sectional studies (*n* = 67) demonstrated robust and consistent associations between higher ‘food responsiveness’ and higher adiposity, and lower ‘satiety responsiveness' and higher adiposity throughout childhood. Children who were more food responsive had higher Body Mass Index *z*-scores (BMIz scores; pooled adjusted *r* = 0.22, *n* = 5707, 7 studies), while those with stronger sensitivity to internal satiety had lower BMIz scores (pooled adjusted *r* = −0.31, *n* = 7140, 8 studies). The same cross-sectional associations were also observed consistently in infancy during the period of exclusive milk-feeding (*n* = 5 BEBQ studies).

An important observation from cross-sectional studies is the clear dose–response relationship between appetite and weight, with a strong graded association from underweight through healthy weight to overweight to obesity, including within the healthy weight range. This indicates that children with obesity (or underweight) do not show abnormalities in appetite, rather subtle differences in appetite may contribute to variation in weight across the spectrum. However, cross-sectional studies cannot shed light on the direction of the relationship, nor the likelihood that appetite plays a *causal* role in weight gain or obesity.

Establishing the *causal* relationship between appetite and weight presents a major challenge. Randomizing children to varying levels of appetite is not possible, so studies must instead rely on naturally occurring variation and take advantage of prospective observational designs. The most informative of these have been bidirectional studies that examine, simultaneously, prospective associations between earlier appetite and later weight, and between earlier weight and later appetite. The first such study, undertaken in young British infant twins from the Gemini cohort (aged 3–15 months; *n* = 2213 infants), observed bidirectionality in the appetite–weight relationship, but with significantly stronger associations from early infant appetite to later weight than from early infant weight to toddler appetite [14]. Findings from *n* = 6 studies of children (aged 4–14 years) are far less clear and appear to vary importantly by adiposity measure [12,13,15,16]. For example, there is evidence of bidirectionality in the appetite–adiposity relationship when adiposity is indexed using fat mass (e.g.[15]). However, studies with BMI, which captures fat and lean mass, show either null bidirectional associations nor a prospective association only from BMI to later appetite. Only one study used BMI observed bidirectionality [17]. These findings are suggestive of developmental variation in the appetite–adiposity relationship such that appetite plays a more important role in adiposity development (than does adiposity in appetite development) in the earliest period of life between birth and toddlerhood, after which the relationship is more complex and may have become too established to disentangle using epidemiological methods. It also highlights the importance of using measures of adiposity that disaggregate fat from lean mass, which is known to be a potent determinant of appetite [18].

However, it is important to note that existing bidirectional evidence is limited: studies have varied in duration of follow-up, age range and frequency of assessment; there is only one study in infancy, no studies have yet examined interactions with infant feeding modality (i.e. breast- and formula feeding), and there are no studies examining the transition from toddlerhood to early childhood; there is also a lack of research in ethnically and socioeconomically diverse samples. Importantly too, few studies have used sophisticated statistical methods that are able to separate out *between-* from *within-person* effects (i.e. how children's appetite/weight changes over time on the group level versus how an individual child's appetite/weight compares to their own appetite/weight across time), which can produce biased or crude estimates. The most major limitation of all epidemiological studies is possible confounding of the appetite–weight association by unmeasured factors such as socioeconomic position, ethnicity, gestational weight gain, gestational diabetes, maternal behaviours during pregnancy (e.g. diet, physical activity or smoking), or parental BMI. Studying prospective weight gain in twin pairs who are discordant for appetite provides a more stringent test of causality than studying the relationship in unrelated individuals, because the co-twin design removes confounding from all possible environmental factors shared completely by twin pairs. The co-twin approach was used in Gemini to compare weight gain trajectories from birth to 15 months for same-sex twin pairs discordant for appetite (by ≥1 s.d.; *n* = 121–172 pairs) in the first few weeks of life [19]. The weight gain trajectories of pairs diverged progressively and significantly over time, such that the twin with the larger appetite (more food responsive or less satiety responsive) gained weight more quickly than his or her co-twin. By 15 months of age there was approximately a 1 kilogram difference, which equated to an approximately 10% difference in body weight within pairs, consistent with early infant appetite playing a causal role in rapid infant weight gain. However, to date, there have been no co-twin studies of the childhood period.

Overall, this research suggests that appetite drives faster weight gain in the early infancy period when the appetite–weight relationship is first becoming established; in childhood, the relationship is less clear and more complex. However, once the appetite–adiposity relationship has been established by early childhood, it may not be possible to make meaningful interpretations about the direction of effects from epidemiological studies, because every increase in weight must be accompanied by an increase in appetite in order to maintain body weight and to grow. It is possible that this process may be too iterative to disentangle cause and effect, even from multiple repeated measures. Other study designs are needed to bring triangulation of evidence, including experimental studies, genetic studies and gene expression studies. Evidence from these other designs is reviewed in the following sections.

### (b) Hypothesis 2—appetite avidity is characterized by distinct patterns of over-consumption in early life

A small but growing number of experimental studies, and behavioural and nutritional epidemiology, have indicated that appetite is expressed as ‘obesogenic’ eating behaviours that, over time, could lead to excess weight gain, and that the predisposition to overfeed begins in early life. The first such investigation, undertaken by Professor Wardle, showed that British children aged 4–5 (*n* = 149) who were more food responsive and less satiety sensitive demonstrated a range of ‘obesogenic’ eating behaviours under controlled experimental conditions at school, including eating in the absence of hunger, impaired caloric compensation, faster eating and greater ad libitum energy intake [20]. Subsequent experimental research has supported these observations [21,22], including a French longitudinal laboratory-based study which demonstrated that toddlers (*n* = 31) who had greater increases in ‘food responsiveness’ from 11–15 months of age also showed greater reductions in caloric compensation across the same time-period [21]. Of particular interest was an experimental laboratory-based study, which found 4–5-year-old non-Hispanic Black US children (*n* = 100) who were less 'satiety responsive' and more 'food responsive' had greater increases in energy intake as portion sizes increased, suggesting that these appetitive traits confer greater susceptibility to overeating in response to a food environment characterized by large portion sizes [23].

To date, only one study has examined how appetite avidity is expressed behaviourally during the very early period of exclusive milk-feeding. Among 3–5-month-old US infants (*n* = 54), those who accepted a second milk feed only 30 min following an initial feed under experimental conditions were rated independently by parents (not in response to the test observations) as having higher ‘food responsiveness’ and lower ‘satiety responsiveness' compared with infants who rejected it [24].

Several epidemiological studies have also examined relationships between appetite and ‘everyday’ patterns of overconsumption characterized as larger ‘meal size’ (more calories consumed per eating occasion) or higher ‘eating frequency’ (more eating occasions per day). Analyses of multiple day food diaries across three independent cohorts found that children who were more food responsive ate more frequently throughout the day; including 21-month-old British toddlers (*n* = 2203) from Gemini [23], 4–7-year-old Portuguese children (*n* = 1359) from Generation XXI [25] and 6–8-year-old Finish children (*n* = 406) from the PANIC study [26]. Analyses of toddlers in Gemini also showed that those who were less sensitive to satiety consumed larger meals but did not eat more frequently [27], suggesting that ‘satiety responsiveness' and ‘food responsiveness’ may have distinct behavioural expressions in toddlerhood. However, in older children from Generation XXI (age 7 years, *n* = 1359), higher ‘satiety responsiveness' was associated with *higher* eating frequency and more daily snacks [25], consistent with the emergence of a ‘grazing’ eating pattern among children with less avid appetites as they get older.

Studies have also examined relationships between appetite and dietary composition, to establish if appetite avidity leads to excessive weight gain partly through differences in diet quality, such as higher energy density. However, findings have not supported this mechanism. On the contrary, some studies have found that higher ‘satiety responsiveness' is associated with *poorer* diet quality—e.g. lower fruit and vegetable intake and a lower Healthy Eating Index [28]. Appetite avidity in early life therefore appears to be expressed by *behaviour* rather than diet quality. In line with this, in Gemini at 21 months of age (*n* = 1939), a larger meal size and more frequent eating were associated with steeper weight gain from 2 to 5 years of age, while diet quality (energy density and macronutrient composition) was not [29].

Taken together, experimental and epidemiological research suggests that appetite is expressed via patterns of overconsumption, rather than dietary quality, during early life. However, it is not entirely clear if ‘food responsiveness’ and ‘satiety responsiveness' are characterized by distinct patterns of overeating and, to date, research in the early period of infancy is lacking.

### (c) Hypothesis 3—appetite has a strong genetic basis and mediates genetic influence on weight in early life

Behavioural genetics studies in infants and children have aimed to establish if: i) appetite, like body weight itself, has a strong genetic basis; and ii) appetite and weight share common genetic aetiology. Much of what we know about the relative contribution of genetic factors to variation in early life appetite comes from twin studies, although there have been relatively few in infants and children. The first such study undertaken in *n* = 5435 pairs of 10-year-old British twins from the Twins Early Development Study (TEDS) reported high heritability estimates for both ‘satiety responsiveness’ (63%) and ‘enjoyment of food’—an appetite trait closely aligned to ‘food responsiveness’ (75%) [30]. A more recent study of a subsample of *n* = 86 pairs of twins from the Generation XXI cohort found heritability estimates of the same magnitude for ‘satiety responsiveness' (88%) and ‘food responsiveness’ (69%), also measured at 10 years of age. The first and only study of the heritability of infant appetite during the period of exclusive milk-feeding, undertaken in *n* = 2402 pairs of twins from Gemini, revealed genetic influence as high as that observed in older children, with heritability estimates of 72% for ‘satiety responsiveness' and 59% for ‘food responsiveness’. Together, these findings highlight that individual differences in appetite have a strong genetic basis from the very earliest period of life, before any solid food has been introduced, to later childhood by which time eating behaviour is highly complex with a multitude of environmental factors at play. However, this research base is small and largely limited to two British cohorts—Gemini and TEDS. Findings need to be replicated in large cohorts from other countries, including non-White European samples and those from low-to-middle income countries.

Twin studies can also estimate the extent of shared genetic aetiology underlying multiple traits (e.g. appetite and weight) using a statistic called the genetic correlation (*r*_G_). It ranges from −1 to +1 and can be interpreted similarly to a Pearson's correlation [31]. To date, only one study has taken this approach in early life, examining shared genetic aetiology underlying appetite and weight at 3 months of age, in *n* = 2402 pairs of twins in Gemini [32]. There were small-to-moderate genetic correlations between weight and ‘satiety responsiveness' (*r*_G_ = 0.23) and a measure of overall appetite size (*r*_G_ = 0.37), although ‘food responsiveness’ was not examined. A few twin studies of adults have also estimated shared genetic aetiology underlying weight and appetitive traits closely related to ‘food responsiveness’, including ‘external eating’, ‘uncontrolled eating’ and ‘disinhibition’ measured using the Dutch Eating Behaviour Questionnaire and the Three Factor Eating Questionnaire. Population-based studies of adults ranging from early adulthood to older age, from the UK, Finland and South Korea, reported genetic correlations between BMI and appetite of a similar magnitude to that observed in infancy (*r*_G_ = 0.25–0.29), although a study of Spanish adult twins during midlife did not find a significant genetic correlation (reviewed in [33]). Overall, these studies indicate overlap in the genetic aetiology underlying appetite and weight, consistent with appetite partly mediating genetic susceptibility to obesity. However, appetite is unlikely to be the only pathway through which genetic influence on weight is expressed, because genetic correlations are only low-to-moderate. To date, there is no research during childhood, which is an important gap that needs to be filled.

Genomic studies have also helped to elucidate the role of appetite in genetic susceptibility to obesity, although there are very few in early life. Studies have examined if: (i) associations between polygenic risk scores for obesity (PRS–obesity), derived from Genome-Wide Association Studies (GWAS), are also associated with appetite; and (ii) appetite mediates the observed association between PRS–obesity (exposure) and measures of adiposity (outcome). The first such study examined the association between a PRS–obesity comprising 28 common genetic variants and ‘satiety responsiveness' in *n* = 2258 10-year-old children from TEDS (‘food responsiveness’ was not examined). Children at higher genetic susceptibility to obesity also had weaker satiety sensitivity, and ‘satiety responsiveness' significantly mediated part of the association between the PRS–obesity and measures of adiposity (approx. 10%) [34]. A follow-up study also found a significant association between a PRS–obesity (comprising 97 genetic variants) and blunted ‘satiety responsiveness’, in *n* = 3016 4-year-old Dutch children from Generation R; however, no association with ‘food responsiveness’ was found [35]. A smaller Norwegian study found no association between an obesity–PRS (comprising 32 genetic variants) with either ‘satiety responsiveness' or ‘food responsiveness’, in *n* = 652 6-year-old children from the Trondheim Early Secure Study (TESS). However, TESS is an unusually lean cohort, and lower variation in BMI may have impacted statistical power in this already small sample [36]. Several large cohorts of adults from across UK, the US, Canada, France and Finland [37–41] have consistently shown evidence of: (i) associations between measured genetic susceptibility to obesity and appetitive traits closely aligned to ‘food responsiveness’ (e.g. ‘external eating’, ‘uncontrolled eating’ and ‘disinhibition’); as well as (ii) mediation of the obesity–PRS and adiposity association by these appetitive traits [37,38,40,41]. Of particular interest was a longitudinal study of *n* = 2464 British adults from the Whitehall II cohort, which found that ‘disinhibition’ mediated 34% of the association between a PRS–obesity (comprising 97 genetic variants) and 20-year BMI trajectories during midlife. This emerging body of research points towards appetite as a neurobehavioural mediator of genetic susceptibility to obesity, although major gaps remain: there are few studies in childhood and none in infancy; and there are no studies in non-White European populations nor low-to-middle income countries [37].

Lastly, gene expression studies of common genetic variants identified in GWAS of adiposity have shed light on potential gene mechanisms [42]. Expression of many of the common genetic variants is enriched in areas of the brain involved in the regulation of both homeostatic and hedonic aspects of appetite regulation centrally, including the hypothalamus, pituitary gland, hippocampus and limbic system. Other cognitive processes may also be involved, such as learning, cognition, emotion and memory. While it is not possible to draw conclusions about links between areas of gene expression and likely behavioural pathways, these studies point towards appetite as a likely neurobehavioural mediator of genetic susceptibility to obesity, together with the wealth of other study designs reviewed.

### (d) Hypothesis 4—there is gene–environment interplay in the development of weight in early life

**Figure 2.**
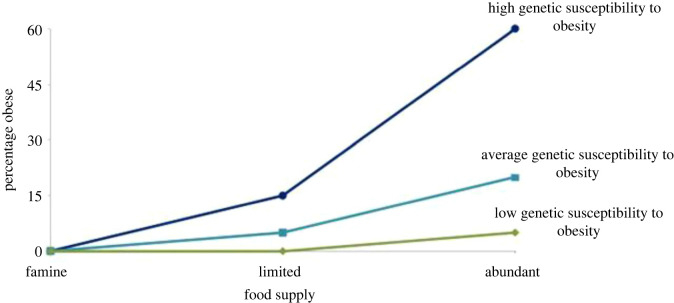
From Llewellyn & Wardle [43]: A hypothetical demonstration of the percentage of children with obesity under three different environmental conditions, according to low, average or high genetic susceptibility to obesity [43].

Central to behavioural susceptibility theory is the hypothesis that genetic influence on weight development depends on the opportunity to eat, and genetic predisposition to obesity will be most fervently expressed in an ‘obesogenic’ food environment. Figure 2 depicts how population rates of obesity would be expected to change in response to different food environments, according to varying levels of genetic susceptibility to obesity. In the context of a famine, no-one develops obesity regardless of genetic risk. In environments with a limited food supply, such as in the U.K. during and immediately after World War II, there will be observable variation in weight, but most of the population will remain lean and genetic susceptibility to obesity is still, largely, buffered. However, in an abundant food supply such as the modern food environment, variation in weight is large as genetic predisposition to higher or lower weight is maximally expressed.

In support of this hypothesis, twin and genomic studies have indicated that genetic expression on childhood weight is more strongly expressed in samples exposed to more ‘obesogenic’ environments indexed at the macro-level (e.g. Western versus non-Western countries), micro-level (e.g. socioeconomic position) as well as the more proximal level (e.g. the home environment). Two large studies of *n* = 45 [6] and *n* = 40 [44] twin cohorts from all over the world found that genetic variation in child and adult BMI was highest in North America and Australia, intermediate in Europe and lowest in East Asia, consistent with decreasing genetic variance from the most (North America and Australia) to the least (East Asia) ‘obesogenic’ environments. Of particular importance, no differences were observed in genetic variances for height, despite mean differences across regions, showing that this effect was specific to BMI [45].

There is also evidence that twin-estimated genetic influence on child BMI is affected by more micro-level factors including family socioeconomic circumstances. In high income, Western countries, neighbourhood deprivation is a marker of a more ‘obesogenic’ food environment [46,47]. For example, in England, there is a strong association between neighbourhood deprivation level and the number and density of fast-food outlets [48]. At the same time, children living in the most deprived neighbourhoods are more than twice as likely to have obesity as those from the least deprived areas [1]. The stark socioeconomic gradient in childhood obesity may therefore reflect, to some extent, exposure to more ’obesogenic’ wider environments, which encourage the behavioural expression of a more avid appetite [47]. In line with this, a large study of *n* = 29 twin cohorts from across the world found genetic variation in child BMI was higher among children whose parents had lower educational attainment [49]. Evidence from genomic studies corroborates that from twin studies insofar as the association between measured genetic risk of obesity and adiposity is stronger among children and adults from a lower socioeconomic background [50,51]. Taking a slightly different approach, a prospective study of *n* = 941 children from Gemini found that those from a lower socioeconomic background had greater increases in ‘food responsiveness’ from toddlerhood to early childhood [52]. Together, these findings support BST's hypothesis that the behavioural expression of an avid appetite is greater when nurtured by an ‘obesogenic’ environment.

The most direct test of gene–environment interaction in the heritability of child BMI was undertaken in *n* = 925 4-year-old twin pairs from Gemini. The families' homes were characterized as either ‘obesogenic’ or ‘healthy’ using the comprehensive Home Environment Interview [53], and the heritability of children's BMI was compared across the two home environments. The heritability of BMI in the ‘obesogenic’ home environments was more than double that in the ‘healthy’ home environments (86% versus 39%), consistent with the hypothesis that obesity-related genes are more strongly expressed in more ‘obesogenic’ home environments and, promisingly, that genetic predisposition to obesity in early childhood could be buffered by a healthy home environment [54].

## 4. Implications, gaps and new directions

The BST offers a conceptual framework for understanding how genes and the environment conspire to determine obesity risk, with biology—expressed as eating behaviour—playing a central role. It challenges weight stigma, and provides insights that have the potential to improve obesity public health policies and weight management interventions.

The prevailing causal model of obesity is one of personal responsibility—that is, weight is a reflection of choices regarding what, how often and how much to eat, and whether or not to engage in physical activity. This leads to over-simplistic, ineffective solutions, e.g. the common mantra ‘eat less; move more’. The notion that weight is entirely within an individual's control leads to weight stigma—negative attitudes towards and beliefs about others because of their weight, such as people with obesity are ‘lacking in self-control’ and that poor parenting causes childhood obesity. However, BST highlights that individuals who are at high genetic risk of obesity will find it hard to resist the urge to eat if food is readily available and promoted aggressively, or to eat only a small proportion of food, when portion sizes are larger than needed.

A survey of *n* = 333 policymakers worldwide reported that more than 90% believe that personal motivation is a strong or very strong influence on obesity [55]. This has resulted in focus on ‘down-stream’ obesity public health policies that place high demands on individual agency (e.g. provision of healthy eating information) rather than ‘up-stream’ policies that shape the choices available to individuals (e.g. through mandatory population-level fiscal and regulatory measures that control the food environment), and are therefore unlikely to be effective. For example, in England, between 1992 and 2020, there have been 14 obesity government strategies and nearly 700 wide-ranging policies, with the majority being highly individualistic [56]. It is therefore unsurprising that none have succeeded in reducing obesity in the population. However, more recently, U.K. policies have shifted towards targeting the food environment—e.g. the 2018 sugar levy, a proposed ban on TV advertising of food high in sugar, fat and salt before 9 pm (to protect children), and regulation of the positioning and promotion of these foods in supermarkets. BST would hypothesize that these newer polices are more likely to be effective than previous ones that relied largely or entirely on individual motivation and behaviour.

Weight stigma is also directed at parents, who are often blamed when a child develops weight problems. However, BST highlights that childhood obesity is far more complex than nurture alone. Babies are not born on a ‘level playing field’; some have a more avid appetite and are more demanding regarding milk feeds and food, and these infants and children present considerable feeding challenges for parents. The mainstream advice for parents is to feed responsively (i.e. offer milk or food in response to a child's hunger and satiety cues), which may lead to excessive weight gain for children who are always hungry, or who have weaker satiety signals. However, there is also legitimate concern that overt and excessive restriction of food by parents disrupts a child's ability to learn to self-regulate their food intake, especially in an ‘obesogenic’ environment in which children must also learn how to eat in moderation. Excessive restriction may also lead to a ‘forbidden fruit effect’, such that children develop a strong desire for the foods they are not allowed to eat. At the same time, limit setting is needed for children who are highly food responsive and have low sensitivity to satiety, to prevent or manage obesity. These are real challenges for parents for which there are currently no clear answers. New programmes of research are focusing on identifying feeding practices that are acceptable to parents and feasible to implement, as well as helpful—or at least not harmful—for children with avid appetites (e.g. the APPETItE study: https://www.appetite-research.com/). Covert restriction of food (i.e. where the child is not aware of the restriction) is one such strategy [56].

An example of covert restriction is through food availability in the home, and there is evidence that a healthy early home environment can help to mitigate genetic expression on young children's weight [54]. Features of a ‘healthier home’ include limited availability and visibility of sugar-sweetened beverages and foods high in sugar, fat and salt, as well as greater availability of and access to fruits and vegetables. However, parents may find it difficult to maintain a healthy home food environment if their child has an avid appetite and pesters them to buy their favourite foods and make them readily available. In support of this, mothers participating in a childhood obesity prevention intervention were less likely to limit their child's exposure to unhealthy foods if they had low sensitivity to satiety [57]. Furthermore, children who were highly food responsive and less sensitive to satiety had poorer weight maintenance following behavioural weight loss treatment. Child appetite can therefore make it challenging for parents to implement obesity prevention and management interventions successfully [58]. Assessment of child appetite prior to an intervention could help to inform the most appropriate intervention type and tailor it, as well as aid our understanding of the role of appetite in determining the large variability observed in intervention outcomes. Development of an appetite screening tool from the BEBQ/CEBQ would make it possible to identify children at high risk of obesity before it develops, allowing for early preventative interventions. However, the value and feasibility of such a tool would need to be determined through research with potential users, including caregivers and healthcare providers.

BST proposes that poor appetite and low interest in eating play a role in the development of underweight and weight faltering, at the other end of the weight spectrum to obesity and rapid weight gain. Low weight and slow weight gain in the infancy and early childhood period can be a cause of considerable anxiety for caregivers and, if severe and prolonged, can cause health problems for the child. As with childhood obesity, BST highlights inherited variation in the neurobiology of appetite as a key causal factor, over and above poor parenting. Historically, the spotlight has been on fussy or picky eating as an important determinant of underweight in early life, but it has not been found to be a reliable predictor [13]; on the other hand, poor early appetite does appear to be important. Cut-off scores have already been derived for the ‘food fussiness’ scale of the CEBQ to identify, with high sensitivity and specificity [59], children with clinically significant selective eating. Similar work could be undertaken for the ‘satiety responsiveness' and ‘food responsiveness’ CEBQ scales to screen for infants and toddlers at high risk of underweight and weight faltering. Of note, the CEBQ scale ‘food fussiness’ has high heritability estimates of the same order of magnitude as those observed for ‘satiety responsiveness' and ‘food responsiveness’, indicating that a broad range of eating behaviour phenotypes are under strong genetic influence (reviewed in [60]).

There is also scope to expand BST to eating disorders, which share many features with obesity (and underweight): eating disorders are moderately-to-highly heritable [61] and genomic studies indicate a high degree of shared genetic architecture with weight [61,62]; underweight, overweight and eating disorders are all disorders of food intake regulation, and about 30% of adults seeking weight loss treatment [63] also have binge-eating disorder. All of this indicates some shared aetiology and implicates appetite in susceptibility to both obesity and eating disorders. However, we know very little about the early life appetite of individuals who go on to develop eating disorders. Epidemiological research in large prospective cohorts (including Gemini, TEDS and Generation R) is currently underway to understand if early life appetite is associated with eating disorder symptoms in adolescence and adulthood and to examine common genetic architecture underlying the two.

An important limitation in all research relating to BST is that it has been largely undertaken in samples from affluent, high income western countries. More research is needed in socioeconomically and ethnically diverse samples, and countries in transition, to establish the validity of BST in populations with high levels of food insecurity and cultural differences in eating behaviour and parental feeding practices, which might differentially impact appetite development and, in turn, risk of developing obesity. More longitudinal studies are also needed, including in genetically sensitive designs.

## 5. Conclusion

BST hypothesizes that childhood obesity develops from a complex interaction between genetic susceptibility and exposure to an ‘obesogenic’ environment, and that appetite is a behavioural mediator of this gene–environment interaction from the beginning of life. BST has been supported by a large and varied research base, offering strong triangulation of evidence that variation in early appetite: (i) is highly heritable; (ii) is associated with patterns of overconsumption; (iii) predicts prospective weight gain; and (iv) shares common genetic influence with weight. Infants and children with an avid appetite are vulnerable to overeating and developing obesity in an environment where food cues are pervasive and opportunities to eat are plentiful; while those who have a small appetite and low enthusiasm for eating are unlikely to develop obesity regardless of the food environment they are exposed to. BST challenges weight stigma, and points towards the importance of ‘upstream’ obesity public health policies that focus on regulating the food environment rather than individual behaviour. More research is needed to establish if and how appetite measures can be used to predict childhood obesity and inform behavioural interventions to prevent or manage it. Including caregivers and children in research into the practical applications of BST is essential.

## Data Availability

This article has no additional data.
